# UK medical tourists in Thailand: they are not who you think they are

**DOI:** 10.1186/1744-8603-10-29

**Published:** 2014-05-06

**Authors:** Thinakorn Noree, Johanna Hanefeld, Richard Smith

**Affiliations:** 1 Department of Global Health and Development, Faculty of Public Health and Policy London School of Hygiene and Tropical Medicine, Tavistock Place 15-17, London WC1H 9SH, UK; 2 International Health Policy Program, The Ministry of Public Health, Bangkok, Thailand; 3 Health Systems Economics, Department of Global Health and Development, Faculty of Public Health and Policy, London School of Hygiene and Tropical Medicine, London, UK; 4 Health System Economics & Dean of Faculty of Public Health & Policy, London School of Hygiene & Tropical Medicine, London, UK

## Abstract

**Background:**

Travel for medical treatment is an aspect of globalization and health that is comparatively less understood. Little is known about volume, characteristic and motivation of medical tourists, limiting understanding of effects on health systems and patients. Thailand is amongst a handful of countries that have positioned themselves as medical tourism destination. This paper examines in unprecedented detail volume and characteristics of medical tourists who travel from the UK to Thailand for treatment.

**Methods:**

As part of a wider medical tourism study, authors gained access to over 4000 patient records from the five largest private hospitals in Thailand. These included information on country of origin, gender, age, arrival month, hospitalization, diagnosis, procedures, length of stay, medical expenditure and type of payment. Patient records were analysed to understand who travels and findings were triangulated with data from the UK International Passenger Survey (IPS).

**Results:**

104,830 medical tourists visited these hospitals in Thailand in 2010. While patients originate all over the world, UK medical tourists represent the largest group amongst Europeans. The majority UK medical tourists (60%) have comparatively small, elective procedures, costing less than USD 500. A significant minority of patients travel for more serious orthopedic and cardiothoracic procedures. Data of individual patient records from Thailand shows a higher number of UK patients traveled to Thailand than indicated by the IPS.

**Conclusions:**

Thailand is attracting a large number of medical tourists including larger numbers of UK patients than previously estimated. However, as many patients travel for comparatively minor procedures treatment may not be their primary motivation for travel. The small but significant proportion of older UK residents traveling for complex procedures may point to challenges within the NHS.

## Introduction

Over the past decade there has been an increase in people traveling to access medical treatment abroad, including a greater number of UK residents who travel for treatment. This type of travel – where patients travel to another country with the expressed purpose of accessing medical treatment – is commonly referred to as medical tourism [1]. The rise of medical tourism has been as a result of changes associated with globalization, which have seen the emergence of a global infrastructure, including cheaper flights and greater communication through the internet, which allows providers from one country to market themselves to consumers in another, and greater requirements for out-of-pocket expenditures for healthcare in many countries [2]. Some countries, including Thailand, have marketed themselves as medical tourism destinations, aiming to attract revenue. Thailand adopted a “Thailand: Centre of Excellent Health Care of Asia” policy in 2003, renewed in 2012, and is now one of the most popular destinations for medical tourists. Together with Singapore and India the country now accounts for an estimated 90% of medical tourism in Southeast Asia [3].

However, despite incidental evidence of individual cases [4] and a growing literature on the topic, empirical information about patients (who they are, their expenditure and the types of treatment they seek abroad for instance) is limited [1,5,6]. A number of studies have focused on the medical tourism industry and examined specific aspects of this, including communication and marketing [2,7,8] and practices of different kinds of providers of services [9,10]. Yet, information about the effects of such travel on individuals and recipient health systems is still limited [5,11-13]. In the small number of cases where data of the macro level impact of medical tourism on health systems or a countries’ economy exists (mainly for Tunisia, countries in the Middle East, Thailand and the UK [3,14-16]), it is not linked to individual patient records. This limits more detailed analysis on the typed of procedures for which patients travel, their country of origin and health and tourism expenditure. Literature focused on the experience of medical tourists on the other hand often focuses on a small number of individual case studies, exploring specific push and pull factors or the patients’ experience [17-20], rarely linking individual experiences to systems’ effects. This paper addresses this gap. It presents the first analysis of a large dataset of medical tourists in five Thai private hospitals, analyzing in depth the travel for medical treatment from one country – the UK - to comprehensively understand medical travel between two countries. It builds on prior work conducted which examined the systems level impact of medical tourism on the Thai health system [3,21] and it extends this through the analysis of patients procedures. Analysis presented focuses solely on patients from the UK as these emerged as the largest group of Western patients in Thailand, and the authors had conducted prior research on outbound UK medical tourism. This paper provides insights into the largest cohort of UK medical tourists to date. Data is analysed with reference to UK patients’ characteristics, procedures and medical expenditure. Insights into patients’ characteristics and motivations are discussed as are implications for the UK. A brief comparison with data from the UK International Passenger Survey, conducted by the Office of National Statistics, is included in the discussion.

## Methods

This paper presents the first analysis of a study extensively examining hospital data of medical tourists in Thailand, focused on patients from the UK. It relies on patient records from the five private hospitals in Thailand in 2010. Hospitals were purposely selected, with researchers choosing the five hospitals which catered for the largest number of international patients according to a survey of 55 hospitals conducted by the Thai Ministry of Commerce in 2007. These five hospitals combined accounted for 63% of all international patients visiting Thailand, while other hospitals surveyed had a market share of less than 1% each. Three are located in Bangkok and two in high density tourist destinations outside of Bangkok. All hospitals in this study provide highly-specialized tertiary care and different packages of services. Ethical approval for the study was sought and received by the London School of Hygiene Tropical and Medicine and each hospital as well. Confidentiality, record anonymization and data storage were approved by the hospital ethics committee in each of the hospitals studied.

A cross-sectional survey of all medical tourists obtaining medical services in five hospitals was undertaken by analyzing patient records from hospital electronic databases. Expatriates were already excluded in hospital database which identifies them by permanent postal address in Thailand, duration of stay more than six months and whether they work in Thailand. Clinicians in the five hospitals surveyed reported that international tourists who fall ill while on holiday (as opposed to medical tourists traveling with intent to seek treatment) visit the hospital with acute symptoms related to travelling activities, such as common cold, acute diarrhea, sun burn or accident. Based on this information, tourists who had fallen ill while on holiday were excluded using the patients’ diagnosis based on ICD-10 classification. Ten variables in terms of demography and service profiles from each patient record, including (1) country of origin, (2) gender, (3) age (4) arrival month, (5) hospitalization, (6) diagnosis, (7) procedures, (8) length of stay, (9) medical expenditure and (10) type of payment were analyzed. Findings from the analysis of data on UK medical tourists from the five hospitals were then triangulated with analysis of the UK International Passenger Survey (IPS) to better understand overall volume of patients traveling and data.

Analysis of the IPS data 2000–2010 was conducted as part of a larger research project on UK medical tourism, findings of which are published separately [16]. The IPS is conducted by the UK Office of National Statistics (ONS), which randomly surveys a representative sample of passengers entering and exiting the UK. These results are then used by the ONS to estimate tourism to and from the UK. It asks a range of questions of travelers including about demographic factors, origin and destination of passengers and their primary purpose of travel [16]. Passengers leaving the UK to travel to Thailand and stating medical treatment as their primary purpose of travel are thus reflected in the IPS figures. Thus the IPS represents a very different type of data (an estimate) compared to the number of actual patient records surveyed and analysed from Thai hospitals. Authors included the IPS, despite this difference as having access and conducted analysis of datasets from an ‘originating’ country - the UK, and a recipient country – Thailand- is unique in the literature and, crucially, allowed for triangulation of the UK IPS.

## Results

### Key findings from medical records of UK medical tourist in 2010

#### Medical tourism to Thailand in 2010

A total of 104,830 medical tourists visited the five private hospitals surveyed in 2010. Most of these visited the hospital more than once – these patients accounted for 324,926 separate visits. Medical revenue generated for the hospitals from these patients was 180 million USD.

These patients originated from all over the world, with the majority – approximately 40% - from the Middle East. These are followed by patients from Southeast Asia, Europe, South Asia and from North America respectively. The high number of patients originating in the Middle East is likely to be because the two largest hospitals included in this study are located in a predominately Middle Eastern neighbourhood in central Bangkok, providing close informal links and advertising. Most patients come from the UAE, followed by Bangladesh and the USA. The largest number of patients from Europe are from the UK (Table 1).

**Table 1:** Top-fifteen countries providing medical tourists to Thailand in 2010

		**Patients**		**Visits**		**Average visits/year**
		**Frequency**	**Percent**	**Frequency**	**Percent**	
**1**	U.A.E.	21,568	20.57	63,457	19.53	2.94
**2**	Bangladesh	8,443	8.05	26,338	8.11	3.12
**3**	USA	7,855	7.49	24,262	7.47	3.09
**4**	Myanmar	7,568	7.22	32,940	10.14	4.35
**5**	Oman	7,096	6.77	21,699	6.68	3.06
**6**	Qatar	5,212	4.97	17,784	5.47	3.41
**7**	**United Kingdom**	**3,935**	**3.75**	**10,779**	**3.32**	**2.74**
**8**	Other African countries	3,857	3.68	17,491	5.38	4.53
**9**	Cambodia	3,837	3.66	10,919	3.36	2.85
**10**	Australia	3,360	3.21	10,136	3.12	3.02
**11**	Kuwait	3,159	3.01	11,330	3.49	3.59
**12**	Japan	1,995	1.90	4,681	1.44	2.35
**13**	France	1,742	1.66	4,275	1.32	2.45
**14**	Germany	1,545	1.47	3,780	1.16	2.45
**15**	Canada	1,474	1.41	4,115	1.27	2.79

#### UK Medical tourists to Thailand

##### Demographic profiles

In 2010, almost 4,000 UK patients travelled to Thailand to access medical treatment (Table 1), accounting for 3.75% of medical tourists and approximately 11,000 visits.

Of UK patients 69% are male and 31% are female (Table 2) and the largest group (26%) are between 45–54 years old, with the average age of a patient being 46.52 years. Male patients are older than female ones, being 48.2 and 42.7 years old respectively. It is noteworthy that almost 11% of patients are aged over 65.

**Table 2:** Age distribution of UK patients

		**Gender**		**Total**	**%**
		**Male**	**Female**		
**Age group**	Less than 25 years	149	172	321	8.16
	25-34 years	299	199	498	12.67
	35-44 years	561	258	819	20.83
	45-54 years	724	291	1,015	25.81
	55-64 years	630	200	830	21.11
	More than 65 years	338	111	449	11.42
	**Total**	**2,701**	**1,231**	**3,932**	**100.00**

Patients from Europe, including the UK, tend to visit Thailand between November and March, pointing to the seasonal nature of medical tourism (ie during the cold months of Europe). In addition, during the 2010 political conflict in Thailand [22], a clear drop in patients visiting for treatment was evident in the hospital’s patient data, but this recovered quickly once the unrest was resolved.

##### Treatment profiles

The vast majority of UK patients - 93% - accessed treatment as out-patients, with only seven percent requiring hospital admission. For those admitted, data shows 32% stay in hospital for only a day, with 17.81% and 14.89% being admitted for two and three days respectively; i.e. 65% of those requiring in patient care do so for less than three days (Table 3). However, around 10% of those required hospitalized for more than two weeks, with 5% of patients staying more than 30 days. 60% of patients were men and 40% women.

**Table 3:** Length of stay

	**Number of admissions**	**%**
1 day	219	31.97
2 days	122	17.81
3 days	102	14.89
4-7 days	121	17.66
8-14 days	53	7.74
15-30 days	35	5.11
31-60 days	33	4.82
**Total**	**685**	**100.00**

### Types of procedure

**Figure 1:**
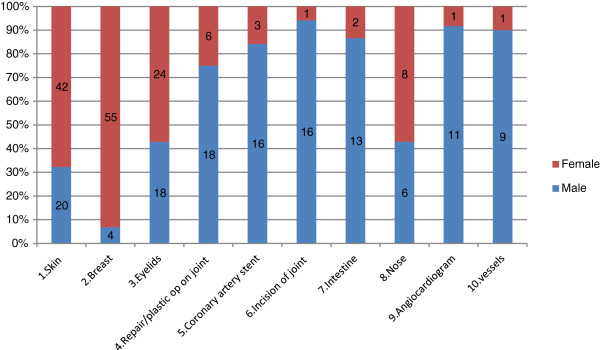
Top 10 procedures among UK medical tourists by gender.

**Figure 2:**
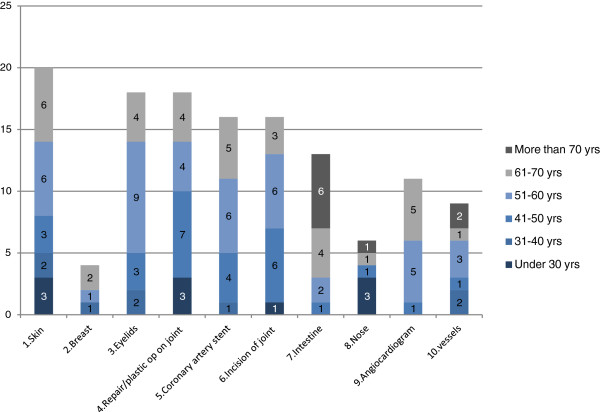
Top 10 procedures among male UK medical tourist by age group.

**Figure 3:**
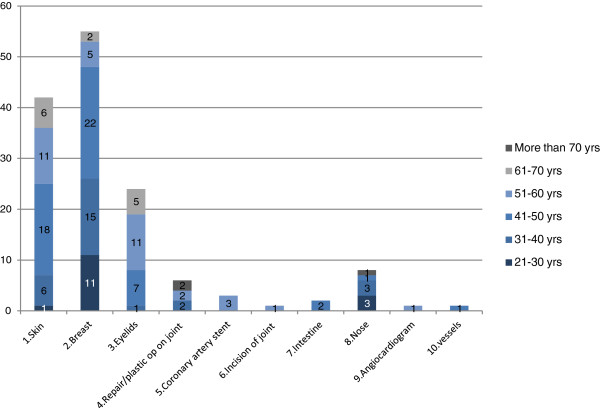
Top 10 procedures among female UK medical tourist by age group.

467 types of procedures were performed on UK patients across the five hospitals during the timespan surveyed, for the purposes of this analysis these were grouped into subcategories of top ten procedures. The distribution of procedures is listed in Table 4. Operations on integument system (mostly breast and facelifts) are the most common procedures, accounting for 25% of total procedures in UK patients, followed by operations on musculo-skeleton, eyes and digestive system respectively (Table 4). Male patients have the major share of all procedures except for cosmetic related procedures (integument, eyes and nose) and those on female genital organs (Figure 1). Women make up the vast majority of patients for cosmetic surgery. There is also a slight age difference between patients; those undertaking cosmetic procedures are on average younger than orthopedic patients (Figures 2 and 3).

**Table 4:** Distribution of procedures in UK medical tourists by ICD-9 classification

	**Type of procedure**	**Total**	**% Total**
1	Operation on the integumentary (mostly breast and facelift) system (85–86)	121	25.91
2	Operation on the musculoskeleton system (76–84)	65	13.92
3	Operation on the eyes (08–16)	55	11.78
4	Operation on the digestive system (42–54)	51	10.92
5	Miscellaneous diagnostic and therapeutic procedures (87–99)	41	8.78
6	Operation on the cardiovascular system (35–39)	21	4.50
7	Procedures and interventions, not elsewhere classified (00) (Mostly coronary artery stent)	19	4.07
8	Operation on the nose, mouth and pharynx (21–29)	18	3.85
9	Operation on the female genital organ (65–71)	16	3.43
10	Operations on the nervous system (01–05)	13	2.78
11	Operation on the urinary system (55–59)	13	2.78
12	Operation on the male genital organ (60–64)	12	2.57
13	Operations on the endocrine system (06–07)	9	1.93
14	Obstetric procedures (72–75)	4	0.86
15	Other miscellaneous diagnostic and therapeutic procedures [17]	3	0.64
16	Operation on the respiratory system (30–34)	3	0.64
17	Operation on the hemic and lymphatic system (40–41)	2	0.43
18	Operation on the ears [18-20]	1	0.21
Total		**467**	**100.00**

### Medical expenditure

The largest group of out-patients (which constitute 93% of all UK medical tourists) - around 40% - pay less than 100 USD per visit (Table 5), with 28% paying between 100 and 500 USD per visit, i.e. almost 60% of patient pay less than 500USD. While 22% of patients pay between 1000 and 5000 USD, most revenue from outpatients is less much less, with average out-patient expenditure at 467 USD (median 160 USD). This contrasts with the small number of in-patients, whose costs are much higher (again as evident in Table 5), with an average in-patient expenditure of 13,955 USD per admission (median 7,522 USD).

**Table 5:** Medical expenditure of UK medical tourists

	**Out-patient**		**In-patient**		**Total**	**%**
	**Number of visits**	**%**	**Number of admission**	**%**		
**Less than 100 USD**	4,034	40.15	0	0.00	4,034	37.45
**100-500 USD**	2,854	28.41	5	0.69	2,859	26.54
**500-**1,000 **USD**	893	8.89	26	3.58	919	8.53
1,000-5,000 **USD**	2,253	22.42	180	24.79	2,433	22.58
5,000-10,000 **USD**	12	0.12	245	33.75	257	2.39
10,000-50,000 **USD**	1	0.01	221	30.44	222	2.06
50,000-100,000 **USD**	0	0.00	39	5.37	39	0.36
**More than** 100,000 **USD**	0	0.00	10	1.38	10	0.09
Total	10,047	100.00	726	100.00	10,773	100.00

## Discussion

Data shows around 4000 UK medical tourists visited the five largest private hospitals in Thailand in 2010. Two thirds of these are men and one third women. This differs from evidence available through the Office of National Statistic’s International Passenger Survey (IPS). According to IPS data in 2010, only 500 UK residents travelled to Thailand for medical treatment. Data from the IPS also indicates that the majority of medical tourists from the UK are women and that over the past decade the largest percentage of travelers have been in the age group of 25–34 year olds. Comparison of hospital data with the International Passenger Survey highlights different specificities of tourists traveling to Thailand (predominately male and older than UK medical tourists traveling to other destinations). The difference between the IPS data and the findings of this survey of UK medical tourists in Thailand are likely due to the differences in methodology used. Yet, it highlights the limits of the information on UK medical tourists available through the IPS.

Our analysis shows that the majority of patients from the UK travel for comparatively low-cost procedures. As 60% of patients’ treatment costs are below USD 500, these were likely significantly lower than the cost of travel to Thailand. This figure points to a significant proportion of people who may simply ‘add’ surgery onto a holiday, or at a minimum have a substantial leisure tourism element as part of their medical travel. It is underlined by the seasonal nature of medical tourism with most patients traveling between November and March, when weather may be a significant factor in patients’ decision to travel to Thailand. The low cost of cosmetic procedures is likely to be a significant motivation. The comparatively smaller amount of resources spent on medical treatment differs from survey results conducted in Europe, where average treatment costs were much higher [23].

Moreover, findings highlight that UK tourists return to Thailand for treatment, and that given the higher number of visits than patients some UK medical tourists may opt for a series of smaller procedures rather than undertake a large procedure.

Despite the majority of patients traveling for out-patient, elective procedures, a number of patients received more complex treatment, such as orthopedic and cardiothoracic procedures, with a small number remaining in hospital for over ten or even 30 days. A comparatively greater number of patients receiving more complex treatment are older. Given the nature of the procedures it is likely that patients would have been entitled to these under the NHS. Further research is needed to understand why these patients may choose to travel.

The idea of a simple ‘add on’ of a small type of treatment is very different to the kind ‘intent for treatment’ which is associated with medical tourism and its definitions in the literature [1]. The mix of patients those with more serious conditions traveling as well as those seeking only smaller types of treatment, suggests that medical tourists are a heterogeneous group.

## Conclusion

Almost 4000 UK medical tourists visit Thailand for treatment annually. While the most popular procedures are elective, UK patients travel for procedures, such as orthopedics and cardiothoracic, for which they would be eligible under the NHS.

Policymakers in the UK need to consider carefully how to reach different groups of patients when thinking about safeguards for UK patients who travel, including in terms of adequate information on quality and risk. Information provided by our analysis also highlights who should receive priority in terms of being targeted with information. While clearly a large number of patients simply travel for small procedures and in particular cosmetic surgery, a small number of older patients travel for more complex orthopedic surgery. This evidence provides an entry point for providing information on safety and risk to UK citizens. The number of patients who travel to Thailand for cosmetic procedures underline the importance of including a focus on medical tourists in the current considerations of regulation of advertising for cosmetic surgery in the UK.

This empirical study adds to the small but growing body of evidence which suggests that medical tourists and medical tourism may be a less unified phenomenon than previously presented in the literature [7].

Finally, a large -scale data set from providers in one medical tourism destination – Thailand – indicates the limitations of current evidence on volume of UK medical tourism available through the IPS. Better data is needed to fully understand UK medical travel and its likely impact on patients and the NHS. Our findings suggest that current data may underestimate the number of UK passengers who travel to access treatment abroad.

## Competing interest

The authors declare that they have no competing of interest.

## Authors’ contributions

TN designed and carried out the data collection and analysis for the study with input from JH and RS. JH and TN provided an initial draft based on the data analysed. All authors reviewed and revised the draft.

## Acknowledgements

TN conceived of the overall study and collected data with input and supervision by RS. TN conducted the analysis with input from JH and RS. JH conducted the analysis of the IPS. All authors met to discuss the findings. TN and JH drafted the initial paper which was revised by RS. This study is part of TN’s PhD, RS is lead supervisor, JH co-supervisor.
